# Targeting LGR5 in Colorectal Cancer: therapeutic gold or too plastic?

**DOI:** 10.1038/s41416-018-0118-6

**Published:** 2018-05-30

**Authors:** RG Morgan, E Mortensson, AC Williams

**Affiliations:** 1 School of Cellular and Molecular Medicine, University of Bristol, Medical Sciences Building, University Walk, Bristol, BS8 1TD UK; 2 School of Life Sciences, University of Sussex, Falmer, Brighton, BN1 9QG UK

**Keywords:** Colon cancer, Colon cancer

## Abstract

Leucine-rich repeat-containing G-protein coupled receptor (LGR5 or GPR49) potentiates canonical Wnt/β-catenin signalling and is a marker of normal stem cells in several tissues, including the intestine. Consistent with stem cell potential, single isolated LGR5^+^ cells from the gut generate self-organising crypt/villus structures *in vitro* termed organoids or ‘mini-guts’, which accurately model the parent tissue. The well characterised deregulation of Wnt/β-catenin signalling that occurs during the adenoma-carcinoma sequence in colorectal cancer (CRC) renders LGR5 an interesting therapeutic target. Furthermore, recent studies demonstrating that CRC tumours contain LGR5^+^ subsets and retain a degree of normal tissue architecture has heightened translational interest. Such reports fuel hope that specific subpopulations or molecules within a tumour may be therapeutically targeted to prevent relapse and induce long-term remissions. Despite these observations, many studies within this field have produced conflicting and confusing results with no clear consensus on the therapeutic value of LGR5. This review will recap the various oncogenic and tumour suppressive roles that have been described for the LGR5 molecule in CRC. It will further highlight recent studies indicating the plasticity or redundancy of LGR5^+^ cells in intestinal cancer progression and assess the overall merit of therapeutically targeting LGR5 in CRC.

## Introduction

Colorectal cancer (CRC) is the third most common malignancy diagnosed globally and the fourth leading cause of cancer-related death worldwide, with its burden predicted to increase by 60% by 2030.^1^ CRC progresses through a well-defined adenoma-carcinoma sequence,^2^ whereby the stepwise acquisition of well-characterised genetic mutations (e.g. *APC, KRAS, TP53*) drives intestinal crypt dysplasia, followed by the development of colorectal tumours. Although detection and treatment protocols have improved markedly, most patients who present with a late-stage cancer will succumb to their disease through relapse. A widely accepted cause of CRC relapse is the failure of current therapies to eradicate cancer stem cell (CSC) subpopulations within a tumour. CSCs express a variety of markers including CD133,^3,4^ CD44, CD166,^5^ ALDH,^6^ EphB2 and leucine-rich repeat-containing G-protein coupled receptor 5 (LGR5).^7^ These CSCs are able to survive therapeutic insult and re-establish tumour growth following therapeutic intervention, and there is therefore an urgent medical need for novel, non-toxic targeted cancer therapies that can induce durable clinical remissions.

A fundamental event for early CRC progression is deregulation of the Wnt/β-catenin signalling pathway, which is constitutively activated through genetic mutations to *APC* or, more rarely, *β-catenin*.^8,9^ Under normal conditions, the absence of a Wnt ligand leads to constitutive phosphorylation of the central mediator, β-catenin, through a destruction complex consisting of GSK3β, CK1, Axin and APC. This primes β-catenin for subsequent degradation by the proteasome, leaving Wnt target genes in a repressed state. Upon Wnt ligand binding to the Wnt receptors Frizzled and LRP5/6, phosphorylated β-catenin saturates the destruction complex but cannot be ubiquitinated or degraded.^10^ The resulting cytosolic accumulation of β-catenin leads to its nuclear translocation where it binds the TCF/LEF family of transcription factors and activates proto-oncogenic Wnt target genes, such as *c-myc*, *cyclinD1* and *survivin*.

**Fig. 1:**
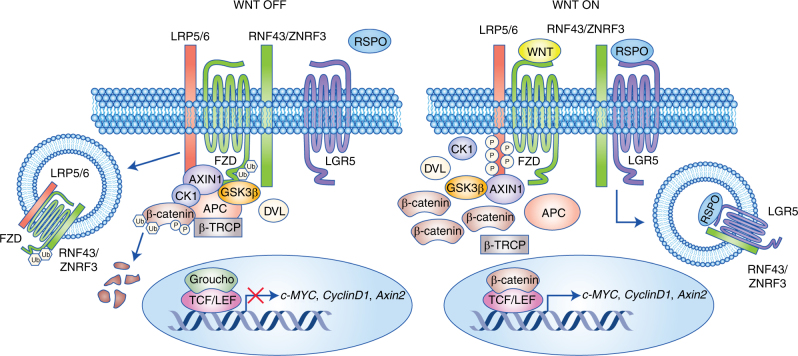
LGR5 promotes Wnt/β-catenin signalling. LGR5 has a well-defined function in the promotion of Wnt/β-catenin signalling in normal intestinal stem cells. Without RSPO bound to LGR5, Wnt signalling is kept low through the action of transmembrane E3 ligases RNF43/ZNRF3, which internalise and degrade the Wnt receptors Frizzled and LRP5/6. This leads to downstream β-catenin degradation and subsequent repression of Wnt target genes. The binding of RSPO to LGR5 sustains Wnt signalling by neutralising the RNF43/ZNF3 ligases, which can no longer remove Wnt receptors from the cell membrane. FZD and LRP5/6 are free to bind Wnt ligands leading to stabilised β-catenin and downstream activation of Wnt target genes such as *c-MYC, CyclinD1* and *Axin2*

LGR5 (also known as GPR49) is a seven-transmembrane protein of the class A Rhodopsin-like family of GPCRs. Within this GPCR superfamily LGR5 falls within the orphan subgroup of glycoprotein receptors (including LGR4 and LGR6), which are characterised by uniquely large ectodomains containing 17 leucine-rich repeat sequences.^11^ Despite its historic classification as an ‘orphan’ receptor, LGR5 now has a well-established ligand in R-Spondin (RSPO), which, when bound, acts in cooperation with Wnt receptors (Frizzled and LRP5/6) to potentiate Wnt/β-catenin signalling.^12–14^ The LGR5/RSPO complex is able to promote Wnt signalling through the neutralisation of two transmembrane E3 ligases, RNF43 and ZNRF3 (Fig. 1).^15^ These enzymes are Wnt targets that remove Wnt receptors from the cell surface, thus serving as part of a negative feedback loop to regulate Wnt signalling output.

*LGR5* is also a target gene of Wnt^16^ and marks normal stem cells in multiple tissues, including the small and large intestine.^17^ The expression of LGR5 during normal intestinal homeostasis is restricted to the stem cell compartment located at the crypt base. This LGR5 expression is lost from stem cell progeny as they migrate upwards through the transit amplifying zone and undergo differentiation.^16^ In support of this, single isolated LGR5^+^ cells from the gut form self-organising crypt/villus structures termed organoids, which are able to recapitulate the full repertoire of differentiated epithelial lineages present in the intestine.^18^ Further studies have shown that stem cell/progenitor hierarchies are maintained in CRC tissue, and that LGR5 acts as a CSC marker.^7,19–21^ This has elevated translational interest in LGR5, since therapeutic targeting of the molecule, or the tumour subpopulations it marks, may represent an efficacious strategy for eradicating tumours and their relapse clones. However, it is over 10 years since the initial characterisation of LGR5 as an intestinal stem cell marker,^16^ and LGR5−targeted therapies have not yet reached the clinic for CRC. Furthermore, the associated literature contains conflicting and contradictory results (Table 1). This review will discuss the various oncogenic and tumour suppressor roles previously ascribed to LGR5 in CRC. We will also recap more recent data highlighting the plasticity and redundancy of LGR5^+^ cells during tumour progression (Table 2

**Table 1:** Summary of the various oncogenic and tumour suppressor roles previously ascribed for LGR5 in CRC

Main findings	Models and studies
*Oncogenic roles for LGR5 in CRC*	
LGR5 is overexpressed in CRC	Primary human/mouse CRC and adenoma cells^23–37^
LGR5 expression predicts adverse prognosis	Primary human CRC;^7,24,26,27,29^ Meta-analyses^41,42^
LGR5 knockdown reduces proliferation, growth, migration, clonogenicity, invasion, PGE2-mediated survival and increases apoptosis, chemosensitivity	Human CRC cell lines;^34,46–48^ Human adenoma cell lines^50^
LGR5 overexpression increases proliferation and chemo-resistance	Human CRC cell lines^27^
LGR5 positivity confers greater clonogenic capacity	Human CRC cell lines and primary human CRC tumours^49^
*Tumour suppressor roles for LGR5 in CRC*	
Loss of LGR5 expression during CRC progression	Human CRC cell lines and primary CRC tumours;^50,53^ Primary human CRC cells^52^
LGR5 expression predicts favourable prognosis	Primary human CRC^52^
LGR5 suppresses Wnt signalling	Mouse small intestine;^54,55^ Human CRC cell lines;^56^ Human CRC cell lines and primary human CRC tumours^57,58^
LGR5 knockdown increases invasion, growth, proliferation (including EGF-mediated) and tumourigenicity	Human CRC cell lines;^56^ Human adenoma cell lines^51^
LGR5 overexpression reduces proliferation	CRC cell lines;^56^ Human CRC cell lines and primary human CRC tumours^57,58^

**Table 2:** Summary of the various studies demonstrating plasticity of LGR5^+^ cells in both the normal gut and CRC

Main finding	Models and studies
Normal gut homeostasis upon loss of LGR5^+^ cells	Mouse intestine/organoids^60–64^
LGR5^+^ pool contains heterogeneous subpopulations	Mouse intestine *in vivo/*organoids^65,66^
LGR5 expression has no prognostic value	Primary human CRC tumours^31,68^
LGR5^−^ cells can sustain tumour growth (LGR5^+^ cells required for metastatic progression)	Human CRC organoids;^21^ Mouse organoids^69^
APC deletion in LGR5^−^ cells is tumourigenic	Mouse intestine/organoids^64^
LGR5^+^ cells interconvert with LGR5^−^ cells for drug resistance	Human cell lines derived from xenografted primary CRCs^70^

), and consider the overall therapeutic merit of targeting LGR5 in CRC.

## Oncogenic roles for LGR5 in CRC

Given its well documented role in potentiating Wnt/β-catenin signalling (and the wider involvement of this pathway in CRC pathology), it is not surprising that many reports have indicated a pro-oncogenic role for LGR5 in CRC. These include studies that have examined LGR5 expression in CRC patient tissue, its prognostic value, and its functional significance in experimental models.

### LGR5 is overexpressed in CRC

The majority of the observational studies in primary clinical tissue have suggested a positive role for LGR5 expression in CRC progression (reviewed elsewhere^22^). Many studies have noted a higher expression of LGR5 in CRC cells, relative to the adjacent normal tissue.^23–34^ Furthermore, a number of studies have highlighted increased LGR5 expression at the invasive front of tumours,^24,26,29–32,35^ and distant metastases.^24,26,27,30,31,36,37^ Caution must be taken when inferring functional significance for LGR5 expression at the invasive front of tumours, since this localisation is also shared with nuclear β-catenin.^38–40^ Given that *LGR5* is itself a Wnt target gene this expression pattern may simply mark Wnt signalling activity, rather than a defined functional role within this setting. In support of this, a study by Baker and colleagues noted heterogeneous localisation of LGR5 expression between the serrated (~ 10–20% cases, non-*APC* mutant) and conventional (~ 80–90% cases, *APC* mutant) pathways of CRC, which may reflect the variable Wnt signalling status of these pathologies.^32^

### LGR5 expression predicts adverse prognosis

LGR5 has been assessed as a prognostic indicator or predictor of response to therapy in CRC; most studies indicate that LGR5 expression is associated with poor clinical outcome. In elegant experiments, Merlos-Suarez *et al* used mouse small intestine to generate gene expression signatures for normal intestinal stem cells, based on expression of LGR5 (and EphB2). When these independent gene signatures were examined in a cohort of 340 CRC patients they were found to strongly associate with disease relapse, metastatic progression, and poorly differentiated tumour types.^7^ Two meta-analyses, one by Chen *et al* (covering seven studies, 1883 patients) and the other by Jiang *et al* (covering 12 studies, 2600 patients), associated high LGR5 expression with shorter overall survival (OS) and disease free survival (DFS).^41,42^ These analyses included studies by Wu,^24^ Hsu^27^ and He *et al*,^26^ which all used immunohistochemical measurement of LGR5 expression in primary patient samples to link higher expression with reduced overall survival in cohorts of 192, 296, and 53 CRC patients, respectively. Takahashi *et al*, used qRT-PCR to assess the *LGR5* level and arrived at the same conclusion.^29^ The study by Hsu and colleagues analysed LGR5 expression in the context of treatment response and reported patients with lower LGR5 expression had a better response to 5FU-based therapy.^27^ Similarly, Stanisavljević *et al* noted a longer time to tumour recurrence (TTR) from patients with low *LGR5* mRNA expression in response to fluoropyrimidine-based adjuvant chemotherapy.^43^ A further study by the Lenz group examined the clinical relevance of germline *LGR5* polymorphisms. The authors identified a single nucleotide polymorphism (SNP) in the *LGR5* gene (rs17109924) that was significantly associated with reduced TTR.^44^ In contrast, an allelic variant of the same SNP (rs17109924) predicts better response to 5FU-based adjuvant chemotherapy.^45^ However, neither study demonstrated how the SNP impacted LGR5 expression, which is crucial given that primary colorectal carcinomas harbouring variant *LGR5* genotypes can exhibit significantly lower LGR5 protein expression.^37^

### Pro-oncogenic functional studies

To complement the correlative studies highlighted above, many groups have attempted to characterise LGR5 function in CRC cells through genetic manipulation of the LGR5 receptor in human CRC cell lines; much of this data has indicated a pro-tumourigenic role for LGR5. Hirsch *et al* used siRNA to show that repression of LGR5 in SW480 and HT29 cells resulted in reduced proliferation, migration and colony formation, both *in vitro* and when xenotransplanted.^46^ These effects were particularly marked in the detached spheroid fraction of SW480 cells, which are enriched for stemcell associated genes (including *LGR5*). Lin and colleagues reported reduced cell proliferation and lower expression of APC and β-catenin upon treatment of HT29 cells with LGR5 siRNA.^34^ An LGR5 siRNA approach was also adopted by Hsu *et al*, who observed suppressed cell growth and colony formation, alongside increased apoptosis on LGR5 knockdown.^47^ Furthermore, work by Chen and co-workers, exclusively in the HT29 cell line, showed that LGR5 siRNA had multiple tumour suppressive effects including: inhibition of cell proliferation, reduced secondary tumour sphere formation, induction of apoptosis, enhanced chemosensitivity, reduced invasive capacity and decreased expression of stem cell markers CD133 and CD44.^48^ Another study by Hsu *et al* involved both transient LGR5 knockdown (LoVo and HT29) and overexpression (HCT116 and HT29) in CRC cell lines.^27^ Whereas treatment with LGR5 siRNA repressed cell proliferation, inhibited colony formation, enhanced apoptosis and sensitised cells to chemotherapy, LGR5 induction increased both cell proliferation and chemoresistance.

Moving from cell lines into primary CRC patient samples, the Medema group demonstrated that the LGR5^+^ fraction of tumours exhibited enhanced TCF/LEF activity (a measure of Wnt signalling output) and greater clonogenic capacity (both *in vitro* and *in vivo*), relative to the LGR5^−^ fraction.^49^ Interestingly, the LGR5^−^ fraction retained some clonogenic growth *in vitro*, and reacquired LGR5 positivity a week post sorting, suggesting a level of dependence on LGR5 for proliferation. In the same study, LS174T cells stably overexpressing LGR5 exhibited enhanced clonogenicity, whereas shRNA-mediated knockdown of LGR5 in primary CSC populations completely abolished clonogenic capacity. Finally, many signalling pathways commonly implicated in CRC are known to target LGR5 expression. LGR5 is a well-established target of Wnt/β-catenin signalling,^16^ but evidence from our laboratory has also shown that the PGE2 and EGF signalling pathways can alter LGR5 expression, which impacts the proliferation/survival capacity of human colorectal adenomas.^50,51^ Collectively, the overexpression of LGR5 in primary tissue, association with poor patient prognosis, and the pro-tumourigenic activity of LGR5 in *in vitro* functional studies would indicate a pro-oncogenic role for LGR5 in colorectal tumourigenesis.

## Tumour suppressor roles for LGR5 in CRC

Studies of LGR5 expression in primary tumour samples and of LGR5 function (including the impact on Wnt signalling) have also suggested a potential tumour suppressive function in CRC development.

### Loss of LGR5 expression during CRC progression

Contrary to the studies discussed above, reports have also suggested that loss of LGR5 expression is observed during CRC progression. De Sousa and colleagues performed gene set enrichment analysis in primary CRC tumours derived from good vs. poor prognosis patients.^52^ Surprisingly, high expression of Wnt target genes (including *LGR5*) was associated with a good prognosis, independent of CSC content or nuclear β-catenin level. Wnt target genes were downregulated during CRC progression and several, including *LGR5*, were found to be methylated in both CRC cell lines and primary tumours. Interestingly, re-expression of these genes through the demethylating agent 5-Aza lowered the clonogenicity of cell lines and primary isolated colon CSCs, and supressed tumour growth upon xenografting. On a similar theme, Su *et al* used methylation-specific PCR to analyse the status of the *LGR5* promoter in six CRC cell lines and 169 primary CRC samples.^53^ *LGR5* promoter methylation was completely absent in normal colonic tissue; however, varying degrees of methylation were observed in half of the CRC cell lines examined (HCT116, complete methylation; SW480 and SW620, partial methylation) and 40% of the primary tumour samples, where it correlated with higher tumour grade. Interestingly, any requirement for a reduction in LGR5 expression during the adenoma-carcinoma transition fits with the LGR5 protein expression pattern we have observed from our panel of human CRC cell lines. High LGR5 protein expression was present in all adenoma cell lines (AA/C1, AN/C1, BH/C1, RG/C2) but absent or low in the majority of carcinoma cell lines (DLD-1, HCA7, HCT116, HCT-15, HT29, LS174T, RKO). LGR5 expression returned to high levels in the metastatic cell lines (LoVo and SW620) suggesting that reacquisition of LGR5 expression could be an important event in the metastatic progression of CRC.^50^

### Negative regulation of Wnt signalling by LGR5

The first indication that LGR5 could have potential tumour suppressive effects in the gut came from studies showing a negative influence on Wnt signalling. Garcia *et al* found LGR5 had little impact on Wnt signalling in the developing small intestine, since *LGR5*-null mice exhibited no alterations to cell proliferation, migration, or epithelial differentiation in the gut.^54^ Further molecular analyses within these LGR5^−/−^ animals actually revealed Wnt activation, with significant upregulation of many Wnt target genes including *Axin2*, *Ascl2* and *CD44*. These findings suggested that LGR5 has a suppressive influence over Wnt gene expression. Similarly, the Tchorz group also reported increased *Axin2* mRNA levels in *LGR5* knockout mice.^55^ Furthermore, Walker *et al* created LGR5-silenced and overexpressing LIM1899 and LIM1215 colorectal cell lines, and showed that LGR5 negatively regulated a subset of established Wnt target genes including *LEF1*, *Frz7* and *WISP1*.^56^

A more recent study by Wu and colleagues identified RSPO2 as downregulated in human CRC cell lines and primary tumours, relative to normal colonic mucosa, due to promoter hypermethylation.^57^ RSPO2 overexpression had an inhibitory effect on CRC cell line growth and Wnt signalling output, whilst increasing LGR5 expression. Silencing of LGR5 reversed these effects by restoring the LRP6 phosphorylation and β-catenin accumulation previously inhibited by RSPO2. Conversely, in HEK293 cells, RSPO2 stimulated Wnt signalling, LRP6 phosphorylation, and β-catenin accumulation, which was reversed by stable LGR5 overexpression. Clearly, context is important since transfection of either RSPO2 or LGR5 alone in HEK293 was sufficient to activate the β-catenin reporter, whereas only the co-transfection of both LGR5 and RSPO2 resulted in supressed Wnt reporter activity. The authors showed that this was achieved through RSPO2 interaction with LGR5 and subsequent membrane stabilisation of the Wnt negative regulator ZNRF3. Finally, observations in clinical CRC tissue by Osawa and colleagues correlated Wnt pathway output with the expression of LGR5 isoforms. Three isoforms were frequently observed that were deficient in exon 5 (LGR5Δ5), exons 5–8 (LGR5Δ5–8), or exon 8 (LGR5Δ8), in addition to expression of the full-length protein (LGR5FL).^58^ Cells overexpressing LGR5FL contained less phosphorylated LRP6 and exhibited reduced Wnt signalling output (determined by TOP-flash assay) compared with cells dominated by LGR5 splice variants.

### LGR5 inhibits the proliferation of CRC cells

Some functional studies have indicated that the LGR5 molecule could have a tumour suppressive role in CRC by limiting cell proliferation. In support of this, silencing LGR5 through both shRNA and siRNA approaches in LIM1899 and LIM1215 CRC cell lines increased invasion, anchorage-independent growth, and enhanced tumourigenicity in xenograft experiments.^56^ Conversely, overexpression of LGR5 resulted in augmented cell adhesion, reduced clonogenicity, and attenuated tumourigenicity. Further support for a tumour limiting role was provided by the Wu study which reported that LGR5 overexpression alone (or in combination with RSPO2) was sufficient to inhibit HT29 cell proliferation.^57^ This result would appear to be at odds with the study mentioned previously, where LGR5 overexpression promoted HT29 cell proliferation.^27^ This difference may have arisen through the contrasting LGR5 overexpression systems used between the studies, with the Hsu study employing transient LGR5 overexpression, and the Wu study adopting stable LGR5 overexpression.

Examination of primary CRC specimens within the Osawa study showed that the LGR5Δ5, LGR5Δ5–8, or LGR5Δ8 splice variants appeared during cell cycle progression, whereas LGR5FL was only expressed during cell cycle arrest. Cells expressing LGR5FL therefore had less proliferative ability than cells expressing LGR5 splice variants, and were negative for the Ki-67 proliferation marker.^58^ Interestingly, the presence of LGR5FL made HT29 cells more chemoresistant, and the proportion of LGR5FL-positive cells in clinical samples was enriched post-chemotherapy, suggesting a positive role for LGR5 in survival and drug resistance. Indeed, our most recent study demonstrated that LGR5 silencing significantly enhanced the sensitivity of human adenoma cells to the epidermal growth factor receptor (EGFR) inhibitor gefitinib.^51^ In this study, LGR5 mRNA and protein expression was suppressed during EGF-mediated proliferation of adenoma cells. Taken together, such findings indicate that LGR5 expression may play a key role in mediating the survival and/or proliferative responses of tumours during malignant transformation.

## Plasticity of LGR5^+^ cells in the normal gut and CRC

The conflicting reports of the pro-oncogenic and tumour suppressive functions of LGR5 in CRC may originate from the inherent plasticity of normal stem cells and CSCs. A host of recent *in vivo* studies have nicely demonstrated the ability of LGR5^-^ and LGR5^+^ cells to freely interconvert during both normal gut homeostasis and CRC progression (recently reviewed elsewhere.^59^)

### LGR5^+^ cell plasticity in the normal intestine

In the normal gut, use of a human diptheria toxin receptor (DTR) gene system knocked into the LGR5 locus has demonstrated no perturbation in epithelial homeostasis upon LGR5 loss.^60,61^ Using this system, DTR is expressed solely in LGR5^+^ cells so that upon DT administration, specific ablation of cells expressing LGR5 occurs. In one of these studies, the Bmi1-expressing compartment (representing a reserve stem pool residing just outside the crypt base) expanded and regenerated LGR5 expressing progeny, indicative of a stem cell hierarchy.^61^ Subsequent reports, however, have shown that this regeneration capacity is not restricted to the stem cell compartment, since more differentiated cell types, including enterocytes,^62^ Dll1^+^ secretory cells,^63^ and KRT19^+^ cells,^64^ are capable of de-differentiation and crypt regeneration upon intestinal damage or LGR5^+^ cell ablation.

There is further heterogeneity even within the LGR5-expressing pool itself. Barriga and colleagues recently demonstrated that the RNA-binding protein Mex3a is a marker of slowly cycling normal LGR5^+^ cells with low proliferative capacity, that potentially survive chemotherapy/radiation and replenish damaged epithelium. Conversely, the Mex3a^neg/low^ pool of rapidly cycling LGR5^+^ cells were inherently more chemosensitive.^65^ Similarly, the Winton group have shown that a quiescent LGR5^+^ subgroup exists (in addition to the rapidly cycling subset) that possesses a differentiated secretory signature and is capable of extensive proliferation upon intestinal injury.^66^ These studies therefore suggest that LGR5 function could vary considerably depending on which cell type it is expressed in, and could be functionally dispensable altogether given the ability of LGR5^−^ cells to compensate and regenerate intestinal epithelium.

### Plasticity of LGR5^+^ CSCs

Given recent findings that a degree of normal clonal architecture is retained in CRCs,^21,67^ it is unsurprising that LGR5 plasticity has also been observed in tumours. This may help to explain why LGR5 expression has previously been linked with no prognostic value.^31,68^ Using their CRC tumour organoid library, the Sato group recently demonstrated that CRC organoids (CCO) have preserved hierarchical heterogeneity, as evidenced by inversely correlated LGR5 and KRT20 expression (marking stemness and differentiation, respectively).^21^ Using elegant lineage tracing experiments, they showed that LGR5^+^ CRC cells could form both LGR5^+^ daughters (KRT20^−^) and LGR5^−^ daughters that acquired KRT20 expression. This indicated that these cells had both self-renewal and differentiation capacity, similar to their normal counterparts. LGR5^+^ cell ablation in CCOs (through an inducible CRISPR/Cas9 system) resulted in apoptosis and tumour regression; however, the LGR5^−^KRT20^+^ fraction of CCOs exhibited proliferation competency and could replenish tumour growth through the regeneration of LGR5 expressing cells.

Similar observations were made by Melo *et al*, who generated mouse-derived intestinal tumour organoids mimicking the sequential acquisition of mutations that occur during human CRC progression (*APC, Kras, Trp53, Smad4*).^69^ Using this model, limiting-dilution transplants of FACS-sorted tumour cells showed that tumour-initiating capacity was enriched in the LGR5^+^ tumour-cell fractions; however, LGR5^−^ cells could also regenerate tumours that subsequently contained LGR5^+^ sub-populations. Upon ablation of LGR5^+^ cells from these organoids using the DTR system, tumours regressed but were not eradicated owing to the presence of proliferating LGR5^−^ cells, which could reform LGR5^+^ CSCs. The LGR5^−^ fraction was not functionally equivalent to LGR5^+^ CSCs because of their inferior capacity to drive tumour growth, and LGR5 positivity was ultimately necessary for metastatic progression of CRC. The same group also demonstrated that the LGR5 protein may even be dispensable for CRC initiation, given the plasticity of the cell of origin in CRC. Whereas previous reports had suggested that tumourigenesis occurred in LGR5^+^ cells at the crypt base,^19^ the study by Metcalfe *et al* found that crypt hyperplasia was unabated upon *APC* loss (a major driver of CRC) in LGR5^+^-depleted mouse small intestines. Rapidly cycling Ki-67^+^ cells were maintained in LGR5−depleted hyperplastic crypts, implying that LGR5^−^ populations could compensate and maintain the proliferative drive.^60^

Along similar lines, Asfaha and colleagues used genetic fate mapping and lineage tracing to characterise two distinct stem cell pools in the mouse intestinal epithelium; the rapidly cycling and radiosensitive LGR5^+^ pool at the colonic crypt base, and a slower dividing, long-lived, radioresistant LGR5^−^KRT19^+^ fraction outside the crypt zone (capable of regenerating LGR5^+^ crypt base columnar cells).^64^ Using targeted *APC* deletion in the KRT19^+^ fraction they were able to show this pool could also serve as the initiating pool in intestinal tumourigenesis distinct from the LGR5^+^ population – a key sign of plasticity.

The modulation of LGR5 expression by CSCs could also be important for mediating drug resistance. Kobayashi *et al* generated cell lines *in vitro* from primary xenografted CRC tumour samples and found that LGR5^+^-sorted cells more efficiently formed colonies in Matrigel and tumours *in vivo*, compared with LGR5^−^ cells.^70^ However, following exposure to drugs such as irinotecan, 5FU, or oxaliplatin, LGR5^+^ cells converted to drug-resistant LGR5^−^ cells with reduced proliferative capacity. Further analysis of these cells revealed that they retained expression of multiple stem cell markers including CD133, CD44, and CD166, but with selectively reduced LGR5 expression. Upon removal of the drugs and reseeding of cells, LGR5 expression was reacquired and proliferative potential restored.

Collectively, these studies suggest that, similar to normal gut homeostasis, there are stages of tumour growth where the LGR5 molecule is functionally redundant. However, the ability of LGR5^−^ CRC cells to rapidly reacquire LGR5 expression implies there are specific phases of tumour growth where LGR5 is important, which is evident during metastatic progression and drug/radiation exposure.

## Other factors affecting LGR5 function in CRC

**Fig. 2:**
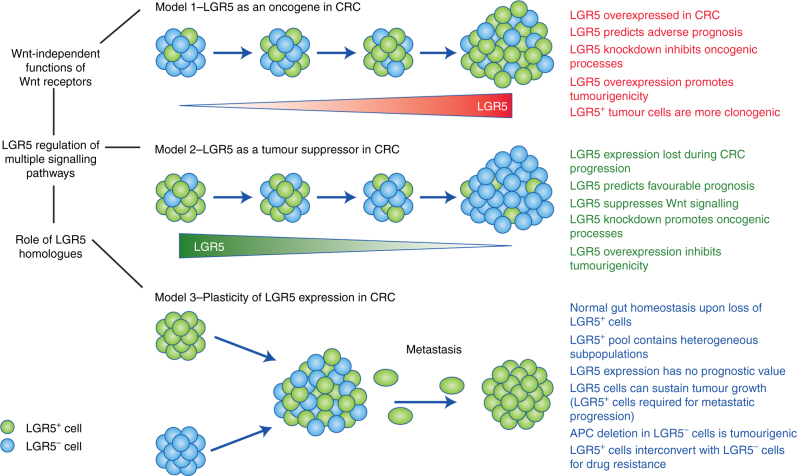
The complexity of LGR5 function in CRC. Model 1: LGR5 is overexpressed in CRC and plays an important role in CRC progression, where it is associated with many oncogenic functions in CRC (in red) and predicts poor outcome. Model 2: Loss of LGR5 expression is important for CRC progression because it performs multiple tumour suppressor roles (in green), suppresses Wnt signalling, and predicts favourable outcome. Model 3: LGR5^+^ cells can freely interconvert with LGR5^−^ cells to drive both oncogenic and normal processes in the gut (in blue). This implies the LGR5 molecule has both important and redundant roles in CRC initiation and progression. A role for the LGR5 molecule in metastasis is likely, given the requirement of LGR5^+^ cells for metastatic progression. LGR5 function in CRC is confounded by multiple variables (in black).

The difficulty in assigning a definitive role for LGR5 in CRC may arise from a number of confounding variables including the alternative functions of Wnt receptors, the effect of LGR5 on multiple signalling pathways, and the role of LGR5 homologues (Fig. 2).

### Wnt-independent functions of Frizzled and LRP receptors

Given that the majority of human CRCs contain *APC* or *β-catenin* mutations, and thus exhibit constitutive activation of the Wnt signalling pathway downstream of the LRP/Frizzled receptors, one might predict LGR5 modulation (and subsequent stability of Wnt receptors) to have negligible effects upon the behaviour of tumour cells. In reality, this now appears to be an overly simplistic view. Recent evidence has demonstrated novel mechanisms of Wnt signalosome regulation by APC, and alternative functions for the Wnt receptors beyond Wnt signalling. Saito-Diaz *et al* recently showed that inhibition of LRP6 (by siRNA and blocking antibodies) was still able to reverse β-catenin signalling in *APC* mutant CRC cell lines, or in cell lines where APC had been deleted.^71^ The Alexander group have demonstrated that LRP5 controls glucose uptake and its depletion suppresses the growth of both normal breast and breast cancer cells (without affecting Wnt signalling).^72^ LRP6 has been shown to interact with PDGFβ and TGF-β receptor 1 at the cell membrane, where it serves as a co-receptor for multiple fibrogenic signalling pathways in pericytes and myofibroblasts.^73^ Furthermore, Frizzled 6 has been reported to mediate non-canonical/PCP Wnt pathways and JNK signalling in a variety of developmental and malignant processes.^74^ Therefore, caution must be taken with studies using LGR5 modulation on *APC* mutant backgrounds, since the altered tumour cell behaviour may still be a result of altered Wnt signalling or Wnt-independent functions of Wnt receptors.

### LGR5 regulation of other signalling pathways

The disparate behaviours of *APC* mutant CRC cells upon LGR5 modulation may be a consequence of the ability of LGR5 to regulate multiple signal transduction cascades (in addition to Wnt signalling). For example, LGR5 can also regulate Notch signalling in CRC cells. Hirsch *et al* reported downregulation of Notch signalling components (including cleaved Notch1 and Sox6) upon LGR5 silencing in SW480 cells, and elevated Notch signalling in LGR5^high^ SW480 spheres. Also in CRC cells, LGR5 has been reported to interact and control the IQGAP1–Rac1 pathway.^75^ The authors showed that LGR5 reduces phosphorylation of IQGAP1 at Ser-1441/1443, thus increasing IQGAP1–Rac1 interaction and enhancing cell–cell adhesion via actin cytoskeleton regulation. Stable knockdown of LGR5 in *APC* mutant LoVo cells led to disorganisation of the cytoskeletal structure and decreased cell adhesion, through disruption of E-cadherin/β-catenin interaction.^75^ Outside of CRC, LGR5 expression can impact upon MAPK signalling, which affects the survival of neuroblastoma cells.^76^ LGR5−targeted siRNA treatment of neuroblastoma cell lines resulted in a dramatic reduction of phosphorylated MEK1/2 and ERK1/2, with an increase in pro-apoptotic BimEL. Should LGR5 also regulate MEK/ERK signalling in CRC cells, this could alter tumour behaviour substantially, particularly during the stages of CRC development where a *KRAS* mutation (which constitutively activates MAPK signalling) has not yet been accrued. Finally, LGR5 has also been shown to regulate Hedgehog^77^ and non-canonical Wnt signalling pathways,^78^ both of which are active in colon cells.

### Role of LGR5 homologues

The LGR5 homologues LGR4 and LGR6 are both capable of binding and co-internalising with RSPO to potentiate Wnt signalling.^12,13^ This has led to the hypothesis that functional redundancy or compensatory mechanisms exist between the LGR homologues. Could this explain the disparate roles for LGR5 in CRC? LGR6 mutations have been reported in colon cancer;^79^ however, its expression is limited to a few tissues including hair follicle stem cells,^80^ but not the intestine,^81^ suggesting a lack of biological relevance in this context. LGR4 has an important developmental role, as evidenced by the neonatal lethality of *LGR4*-null embryos,^82^ and it is broadly expressed throughout the intestinal crypt (not restricted to the crypt base stem cell positions like LGR5).^83^ The lack of any overt intestinal phenotype upon conditional deletion of LGR5 has led many to hypothesise that LGR4 could compensate for LGR5 loss in the intestine.^55^ Furthermore, the finding that LGR5 overexpression can recover the lost RSPO-induced β-catenin signalling induced upon LGR4 loss, has led to speculation that LGR4 and LGR5 have overlapping functions.^84^ Like LGR5, LGR4 is also overexpressed in CRC, where it is a poor prognostic indicator, an enhancer of Wnt/β-catenin activity, and increases the invasiveness and metastatic potential of CRC cells.^85,86^ None of the studies discussed above (*See 'Plasticity of LGR5*^*+*^ *cells in the normal gut and CRC*’) have examined any potential interplay between LGR4 and LGR5 in CRC. It would be particularly interesting to see if the conclusions drawn from the Melo or Shimokawa studies (replenishing of tumours by LGR5^−^ cells following LGR5^+^ cell ablation)^21,69^ would be affected by the co-ablation of both LGR5^+^ and LGR4^+^ cells. Given these studies indicating the alternative functions of the Wnt receptors (controlled by LGR5), the regulation of multiple signalling pathways by LGR5, and functional compensation by LGR5 homologues, it is thus challenging to assign a single pro-oncogenic or tumour suppressive role for LGR5 in CRC.

## Targeting the LGR5 protein for CRC treatment

Assuming LGR5 does have an important role in CRC initiation and progression, is the molecule even druggable? The protein satisfies two highly desirable drug-design criteria: it is a GPCR, and it is cell-surface expressed. Yet, despite structural characterisation of LGR5 nearly 20 years ago, no specific pharmacological LGR5 inhibitors are commercially available. Junttila and colleagues obtained encouraging results through the deployment of an antibody-drug conjugate (ADC), involving an LGR5−targeted antibody conjugated to the potent microtubule inhibitor monomethyl auristatin E (MMAE).^87^ LGR5-MMAE was effective at reducing tumour size and proliferation in both xenografts and APC^min^KRas^G12D^ models of intestinal tumourigenesis, prolonging the survival of mice with minimal toxicity in the relatively short time period analysed (16 weeks). LGR5-MMAE also demonstrated efficacy within a primary human pancreatic cancer xenograft model, providing hope that LGR5-targeted ADCs could be adopted in other cancer types.

A study by the Sato group demonstrated some success by combining a more acute targeting of LGR5 in organoids (using an LGR5-iCaspase9 knock-in system) with widely used CRC clinical agents, such as cetuximab (anti-EGFR antibody) and oxaliplatin (platinum-based chemotherapeutic).^21^ Interestingly, cetuximab in combination with LGR5 ablation exhibited the best synergy, in agreement with our recent study that demonstrated increased killing of human adenoma cells when LGR5 knockdown is combined with gefitinib (EGFR inhibitor).^51^ However, in the Sato study, tumour regression appeared to be dependent on the prior ability of the agent to induce LGR5 mRNA expression (which oxaliplatin did not), and CRISPR/Cas9-based therapies currently remain some distance from the clinic.

It is important to note that, in light of recent data demonstrating that cancer and metastasis-driving LGR5^+^ cells can be freely replenished from the LGR5–pool of a colon tumour,^69^ patients would have long-term or even life-long dependence on LGR5-targeted therapies. With this in mind, a thorough assessment of the long-term treatment-associated toxicity of LGR5 targeting is critical given the importance of LGR5 to normal colonic stem cell biology, and the previously reported side effects (severe gut and liver toxicity) of such strategies.^61,87^

Finally, if pharmacological targeting of LGR5 proves to be non-viable, ineffective, too toxic, or expensive then perhaps other avenues for LGR5 targeting in CRC treatment could be pursued. There are a number of studies showing that dietary factors such as high fat,^88^ curcumin,^89^ vitamin D,^90^ and alcohol,^91^ along with alterations to the gut microbiota,^92^ can affect both the frequency and tumourigenicity of LGR5^+^ cells, and LGR5 expression level. Such approaches to modulate LGR5 expression in tumour cells would be cost-effective if they could be proven to reduce CRC incidence or promote tumour regression.

## Conclusions

Studies examining the role of LGR5 in CRC have produced disparate conclusions most likely arising from the breadth of experimental models and systems used, and the intricacies of LGR5’s function. The complexity of CRC tumours, driven by factors such as tumour heterogeneity, tumour stage, cell of origin, stem cell hierarchies and the microenvironment mean that LGR5 function is likely to vary considerably, depending on the context in which it is assessed. Despite the controversy surrounding the role of LGR5 in CRC, it would be hard to argue against an important role for LGR5 at specific points during CRC development or progression. In particular, there seems to be some consensus on a role for LGR5 in the survival and/or metastatic progression of CRC cells. Therefore, the timing of any LGR5-directed therapy will be crucial for its long-term efficacy, and tumour stage-specific assessments of LGR5 therapies would be useful within *in vivo* models of CRC metastases. This would define where LGR5 targeting (individually or in combination with current chemotherapeutics) is most effective, for example at preventing/reverting metastatic conversion. This would circumvent the toxicities associated with long-term LGR5^+^ cell ablation and identify a stage of human CRC development where LGR5 therapies could induce robust clinical remissions.
